# New Horizons for Consumer-Mediated Health Information Exchange

**DOI:** 10.1055/s-0044-1800741

**Published:** 2025-04-08

**Authors:** Prashila Dullabh, Rina Dhopeshwarkar, Priyanka J. Desai

**Affiliations:** 1 NORC at the University of Chicago, Bethesda, MD, USA

**Keywords:** Health Information Exchange, Patient Generated Health Data, Consumer-mediated Exchange, Patient-directed Health Data Exchange, Electronic Health Records, Consumer Health Information

## Abstract

**Objectives** : In this paper, we discuss current trends in consumer-mediated health information exchange (HIE) within the U.S. and globally, including new approaches, relevant standards that support HIE and interoperability centered around the patient, remaining challenges, and potential future directions.

**Methods** : We conducted a narrative review of the peer-reviewed and gray literature to characterize the current HIE landscape in relation to patient-centered data. Our searches targeted literature in three key areas related to consumer-mediated HIE: policy and initiatives, standards, and the technology landscape.

**Results** : We discuss current trends in consumer-mediated exchange within the U.S. and globally, focusing on policies, standards, and technology that support information exchange centered around the patient. We also outline remaining challenges and potential future directions.

**Conclusions** : The current landscape in the U.S. and globally supports a more patient-centered care model. Ongoing advances in technology and data standards provide the technical infrastructure to empower consumers to electronically exchange their information with different stakeholders in ways not possible just a few years ago. These advancements hold great promise for patients to play a more central role in sharing their information in support of more patient-centered care. Additional research and analyzes along with public policies are needed.

## 1. Introduction

Across the globe, the emphasis in healthcare is increasingly on patient-centered care—defined as being “respectful of and responsive to individual patient preferences, needs, and values, and ensuring that patient values guide all clinical decisions” [ 1 , 2 ]. In 2016, the World Health Organization (WHO) developed a framework for integrated people-centered health services and passed a resolution to strengthen people-centered health services [ 3 ]. Progress in both conceptualizing and implementing patient-centered care has been seen in several countries [ 4 5 6 ]. In the United States (U.S.), public policy, regulations, and support have been strong and enduring for the principles that patients should: i) have access to their own electronic health records (EHRs), and ii) be able to share their records with third parties, including researchers [ 7 , 8 ].

Traditionally, care teams, health systems, and policymakers have relied on clinical data (e.g., lab tests, imaging, vital signs) generated during a clinical encounter to inform healthcare decision making. Recognition is growing of the critical role of patient-centered data in understanding the whole person—thereby informing decision-making for patients, caregivers, and care teams [ 9 ]. Five types of patient-centered data, many of which are directly contributed by the patient, are increasingly relevant in supporting patient-centered care delivery:

- Patient-generated health data (PGHD)— e.g., monitoring of sleep, oximetry, weight, heart rate, blood glucose, blood pressure, and physical activity; as well as self-reported symptoms, treatments, and lifestyle factors (e.g., mood, psychological stress, smoking behavior) [ 10 , 11 ];
- Patient-reported outcomes (PROs) data—questionnaire and survey information about the patient experience, such as quality of life and function [ 12 ];
- Patient preferences data—provide the basis for how patients wish to interact with their clinician, care system, or personal data; choose a particular course of action over others; or prioritize services or effects of healthcare [ 13 ];
- Social determinants of health (SDOH) data—social and environmental risk factor data on non-medical factors that may influence healthcare decisions and health outcomes [ 14 ];
- Genetic and genomic data—underlying risk factors, inherited disorders, and complex diseases [ 15 16 17 ].

Patients should be able to electronically share their data with their care team and other stakeholders (e.g., researchers and public health). Consumer-mediated exchange gives patients the ability to aggregate and manage their electronic health information. When in control of their own health data, patients can track and monitor their own health, help transfer their data between clinicians to facilitate care coordination, correct their data, and share their data for other purposes such as research [ 18 19 20 ].

**Figure 1.**
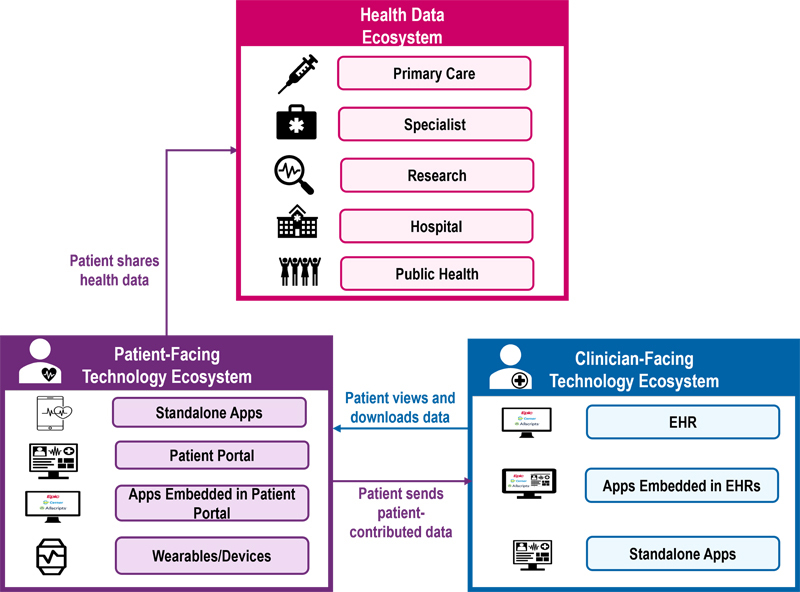
Consumer-Mediated Health Information Exchange: Download and Send Data.

There are currently two ways in which consumer-mediated health information exchange (HIE) can take place. One way is for patients to download their health data and send their information to clinicians, health systems, or researchers using an application (app) or some feature of providers' personal health records (PHRs) or patient portals—which are often tethered to their EHR ( Figure 1 ). An early example of an app that facilitated patients' ability to download and share their health information is the Department of Veteran Affairs' (VA's) Blue Button application within the VA patient portal. The Blue Button enabled VA patients to view, print, and download their medical record data and share those data with non-VA providers [ 21 ].

**Figure 2.**
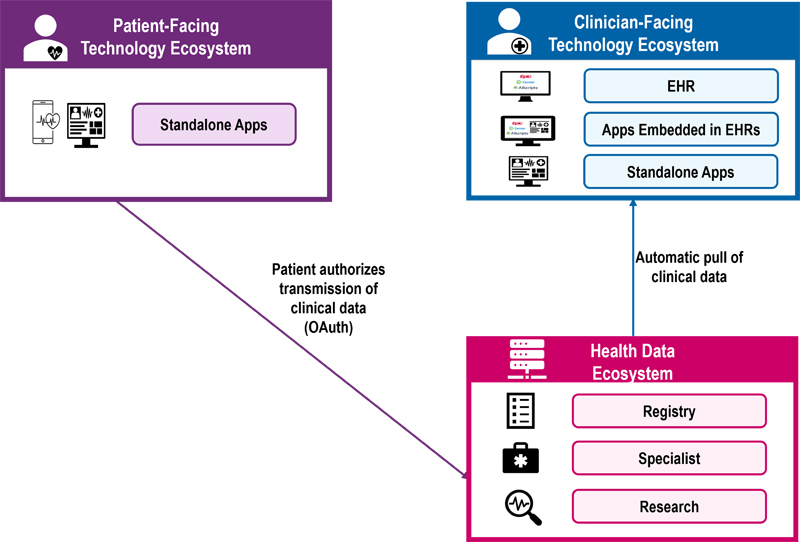
Consumer-Mediated Health Information Exchange: Authorize Direct Transmission.

Another way consumer-mediated HIE can occur is for patients to use consumer-facing apps to authorize direct transmission of their health information from one care organization to another or to a different type of destination such as a researcher ( Figure 2 ) [ 22 ]. For example, Sync for Science facilitates patient-mediated data donation for research use. Upon receiving patient authorization, the requesting organization will pull data from the patient's care provider's EHR and transmit it to a research institution or coordinating center. This approach allows for more passive and continuous data transmission to facilitate consumer-mediated transfer for research [ 23 , 24 ].

The burgeoning app landscape creates new avenues for patients to electronically access and share their data with their care team and others. Apps not only enable patients to view and download their clinical information from health information technology (IT) systems, but also empower patients to directly capture and share PGHD, SDOH, and other data generated outside the clinical encounter. Over 350,000 consumer apps related to health and healthcare are now available [ 25 ]. But embracing patient-contributed data and opportunities for patients to access and share those data raises new considerations for HIE and interoperability [ 26 ].

## 2. Objectives

In this survey paper, we discuss current trends in consumer-mediated HIE within the U.S. and globally, including new approaches, relevant standards that support HIE and interoperability centered around the patient, remaining challenges, and potential future directions.

## 3. Methods

To characterize the current HIE landscape in relation to patient-centered data, we conducted a narrative review of the peer-reviewed and gray literature [ 27 ]. Our searches targeted literature in three key areas related to consumer-mediated HIE: policy and initiatives, standards, and the technology landscape. We searched PubMed, standards organizations' websites, and U.S. federal agencies' websites, and conducted Google searches to identify international regulatory initiatives. We focused on publications within the past 10 years but focused our findings on more recent literature, using sources to provide historical context as needed.

Three questions guided our abstraction and synthesis of 24 peer-reviewed and 45 gray literature sources:

What is the current state of consumer-mediated HIE?

What key challenges need to be addressed to support the exchange of patient-centered data?

What are the short- and long-term opportunities to advance patient-centered HIE?

In our assessment of the technology landscape, we found several articles referencing patient portals and PHRs where they do not make a distinction between the two. Given this, throughout this paper we use the terms patient portal and PHRs interchangeably.

## 4. Results

### 4.1. Policy and Initiatives: Current Landscape of Consumer-Mediated Health Information Exchange

Studies of consumer perceptions of HIE have shown that granting patients greater control over the exchange of their electronic health data can address their confidentiality concerns, by enabling patients to control who can access their health information, and exactly which information they access [ 28 ]. Consumer-mediated HIE can also address challenges in matching patient records across two care organizations trying to directly exchange patient health information. In the U.S., where an individual's health information can be fragmented across multiple health care organizations, consumer-mediated HIE offers a mechanism for consolidating and aggregating data across different provider-maintained EHR systems [ 20 ].

#### 4.1.1. Supportive Policies and Initiatives

Recognizing the importance of allowing patients to access and share their own health information, several countries have implemented public policies, regulations, and government-led initiatives to create an environment supportive of consumer-mediated HIE.

In May 2022, the European Commission published a proposal for the European Health Data Space (EHDS), which aims to improve patient's individual access to and control over their electronic health data and provides a common framework for reuse of health data for research, policy making, regulatory tasks, and innovation including artificial intelligence training and personalized healthcare [ 29 ]. The EHDS enables European Union (E.U.) citizens to allow health professionals across E.U. countries to access their personal health data via a digital interface. In addition to enabling cross-border data exchange, the EHDS establishes a single market for digital health products and services and creates an EHR exchange format with defined data categories for patient summaries and electronic prescriptions [ 30 ]. The provisions set forth for the EHDS may lay the groundwork for other countries to take similar steps toward granting patients' rights over access to their electronic health data for sharing and reuse [ 31 ].

Many other countries have developed processes and systems to allow citizens to access their own patient data [ 32 ]. In 2020, the Global Digital Health Partnership (GDHP) published a white paper on Citizen Access to Health Data. The majority of GDHP participant survey respondents, which included 22 participating countries and territories, reported that electronic access to personal health information is now available to all or most citizen users—including information on medications, immunizations, diagnostic test results, and hospital records. Reportedly less likely to be available are outpatient records, community/primary care records, and summaries of encounters/clinical notes [ 33 ].

While many countries are facilitating patients' access to their own data or the exchange of health data between care organizations, the Netherlands is leveraging interoperability resources to support the exchange of health data between patients and their clinicians or care organizations. MedMij establishes a governance framework, certification, and standards; and delineates roles for both patient-facing and healthcare provider— facing personal health data service providers. These service providers leverage standard-based application programming interfaces (APIs) that enable patients to access their health data in a personal health environment (a digital tool to track health information) of their choice. The Netherlands Ministry of Health subsidizes every patient who uses a MedMij-certified personal health data provider, so patients can use the system for free [ 33 ].

In the U.S., the 21st Century Cures Act requires secure access to patient health information through Health Level Seven (HL7®) Fast Healthcare Interoperability Resources® (FHIR®)-based APIs, with the goal of enabling more patients to access and engage with their health information through apps [ 34 ]. Similarly, the Centers for Medicare & Medicaid Services (CMS) Interoperability and Patient Access Final Rule requires CMS-regulated payers to implement and maintain a secure, standard-based (HL7® FHIR®) Patient Access API [ 35 ]. The 2020 Office of the National Coordinator for Health IT (ONC) Cures Act Final Rule prohibits the act of “information blocking”—the practice of interfering with the access, exchange, or use of electronic health information. To comply with the information blocking regulations, healthcare providers have increased the amount and type of electronic patient health information that patients can access and download from their patient portals [ 36 ].

Together, these efforts create a supportive policy landscape globally to further promote standards development, adoption, and use, and encourage development of technology tools and solutions (such as health apps) that provide patients access to, and use of, their own health data to better support person-centered care. At the same time, other factors still hinder or negatively influence patients' ability to, or interest in, accessing their health data.

#### 4.1.2. Key Challenges

Four key challenges limit the widespread ability of patients to engage in consumer-mediated HIE [ 37 ]. First, it is difficult to semantically integrate information from multiple sources. Even if patients can access medical record information by downloading it through patient portals made available by their healthcare organizations, these records can be cumbersome in length and challenging for patients to parse [ 38 ]. A patient summary standard such as the Continuity of Care Document—which offers a standard structure and semantics for summarizing the most relevant data from a patient's medical record for electronic HIE—can address this challenge, but Continuity of Care Documents are not always available for patient download. Additionally, patients or clinicians would need to integrate Continuity of Care Documents into a single summary.

Second, low adoption rates of health IT among certain healthcare providers and care settings can result in patients having limited access to their electronic health information and/or result in data that have gaps or are generally low in quality. For example, in the US, even though health IT adoption is high among hospitals and professionals eligible to participate in the Promoting Interoperability Program, smaller or rural hospitals and lower-resourced health professionals—which include those practicing in behavioral health, and home and community-based services—do not have any incentives to adopt health IT [ 39 , 40 ]. Globally, health IT adoption varies widely, particularly in primary care settings, and can be constrained by a range of factors including lack of training, costs, and management support [ 41 , 42 ].

Third, even if patients—particularly those managing complex and chronic conditions—do want to access their data, they may lack knowledge about available applications or features of their patient portal that would allow them to access, download, and transmit their own health information [ 43 ]. Usability challenges regarding apps/patient portals can also hinder patients' access to their own health information [ 44 ]. A US-based study of nationwide 2014–2016 hospital data found that healthcare organizations can also influence patients' access to and use of their health information. Compared to patients in hospitals serving underserved populations, patients served by hospitals that were part of health systems had higher levels of electronic health information access and use, patients of teaching hospitals had higher levels of information use, and patients of for-profit hospitals had higher access to information but lower use. This points to the need for policies that encourage healthcare organizations to offer access to and improve patients' use of electronic health information [ 45 ].

Finally, although allowing patients to control the sharing of their own electronic health information avoids barriers related to obtaining appropriate patient consent for HIE, there are other privacy and security concerns to be considered for consumer-mediated HIE. Studies have shown that concerns around privacy may be the principal factor affecting personal health information sharing and that when there is more transparency in data sharing policies patients are more inclined to agree to share their data [ 46 , 47 ]. For the download and send approach, there are potential security risks if healthcare organizations do not have adequate protocols and policies in place for proxy access to patient portal accounts. This includes risks to data confidentiality resulting from sharing of password credentials when proxy account access is not available, and privacy concerns when there are not features available for limiting the type of information accessible and duration of time proxy account holders can access information within the patient portal [ 48 , 49 ]. When patients are authorizing transmission of their health information or directly sharing data, there are important considerations around health literacy, digital literacy, and ensuring that patients are fully cognizant of the specific data they are sharing and should share [ 50 ], the recipients of that data, and how the data are to be used [ 51 ]. Additionally, several studies have found positive associations between health literacy and patients accessing their own data [ 52 ] and their willingness to share their data for clinical or research use [ 53 ].

### 4.2. Developing and Adopting Standards: Supporting Patient-Contributed Data Capture

As interest in and opportunities for consumer-mediated exchange continue to grow, the technical landscape is evolving to support not only the exchange but also the capture of patient-contributed data (e.g., when a diabetic patient wants to share their home blood glucose readings with their clinicians, this would be patient-generated data from a home medical device). Such advancement requires the adoption of semantic and syntactic standards to ensure that patient exchanged data can be integrated into EHRs and other digital health systems. Standards are critical to enable seamless data capture and movement in two-way patient-clinician exchange; among clinicians; and across care organizations in the health data ecosystem. Below, we summarize existing standards and initiatives supporting patient contributed data capture and exchange and key challenges.

#### 4.2.1. Existing Standards and Initiatives

Availability of standards for patient-centered data varies. Data like PROs, SDOH, and genetic/genomic information have established terminology standards (i.e., Logical Observation Identifiers, Names, and Codes (LOINC) and SNOMED Clinical Terms (SNOMED CT)) and are reflected within HL7® FHIR® resources to support capture and sharing [ 54 55 56 ]. But in areas like PGHD (for which the exchange of large volumes of data generated from devices/wearables is challenging) and patient preferences (for which information is often captured as unstructured data), the field is still evolving [ 57 58 59 ].

Globally, interoperability standards such as HL7® FHIR® are increasingly developed and adopted to support the exchange of patient-centered data. The HL7 Gravity Project has developed and is testing data elements and associated value sets to represent SDOH data on food insecurity, housing instability, and stress in screening, diagnosis, goal setting, and interventions [ 60 ]. The SMART Markers software framework supports PGHD exchange from patient apps or devices to the point of care [ 61 , 62 ]. The framework's “Request & Report” model allows clinicians to send PGHD requests to patients via a tablet or an EHR app, and patients to respond to these requests on their devices [ 61 ]. In July 2023, the WHO and HL7 International® signed a Project Collaboration Agreement that will support the use of WHO international classifications and terminologies by the HL7® FHIR® community [ 63 ]. The agreement also allows for HL7® FHIR®-enabled SMART Guidelines to be made available with multilingual support in six official languages of the United Nations: Arabic, Chinese, English, French, Russian, and Spanish. Table 1 provides examples of how the FHIR standard supports the capture and exchange of patient-centered data.

**Table 1.** Examples of FHIR Resources to Support Consumer-Mediated Exchange.

Data Type	Example Applications of the FHIR Standard
PROs	The HL7 ^®^ FHIR ^®^ PRO Implementation Guide focuses on the capture and exchange of PROs data [ 64 ] [ 64 ]. Recent initiatives have used the guide to develop FHIR-based mobile apps for collecting PROs data [ 64 ].
PGHD	Apple HealthKit uses FHIR to support PGHD sharing with Epic's MyChart app [ 10 , 65 ]. CommonHealth leverages HL7 ^®^ FHIR ^®^ and SMART Health Cards to connect users to their EHR data and give them control of how, when, and with whom they share their health data [ 66 ].
Patient Preferences	The FHIR Questionnaire Resource can be used to capture patient-directed questions and the QuestionnaireResponse Resource can be used to capture patient responses to these queries [ 67 ]. The FHIR Goal Resource can be used to capture treatment preference information [ 68 ].
SDOH	The Gravity Project's FHIR SDOH Clinical Care Implementation Guide focuses on documenting and exchanging SDOH data, focusing on three use cases [ 69 ].
Genetic and Genomic Data	The Sync for Genes initiative has explored exchanging genomic test results using the FHIR standard and using FHIR APIs to share patient genomic data [ 23 ].

FHIR standards support HIE across borders. The International Patient Summary (IPS) standard was developed through a collaboration of several standards development organizations—including HL7 International, the European Committee for Standardization (CEN), Integrating the Healthcare Enterprise (IHE), the International Organization for Standardization (ISO), and SNOMED International. IPS is a “minimal and non-exhaustive set of basic clinical data of a patient, specialty-agnostic, condition-independent, but readily usable by all clinicians for the unscheduled (cross-border) patient care” [ 70 ]. Another HL7 initiative arising from the IPS FHIR work is International Patient Access (IPA). IPA provides specifications for how a patient-facing app can access patient health from clinical records systems using a FHIR-based API [ 71 ].

Within the U.S., the United States Core Data for Interoperability (USCDI) provides a core set of standardized health data for interoperable HIE across care settings. USCDI defines a common set of data needed for a wide range of use cases. These standardized data are regularly exchanged within and between certified health IT and are increasingly exchanged within and across other environments (e.g., data registries, data repositories, and consumer-facing apps) [ 72 ]. Recent additions to the USCDI include data elements for patients' treatment intervention preference and care experience preference, as well as SDOH assessment, goals, and concerns [ 73 ].

#### 4.2.2. Key Challenges

Three cross-cutting challenges confront all major patient-centered data types (PROs data, PGHD, patient preference data, SDOH, and genetic/genomic data)—all will require collaboration between standards developers, policymakers, researchers, and patient partners to address. First is the lack of sufficient terminology standards to support structured data capture. SDOH data may be captured in unstructured clinical notes within the EHR, making data exchange hazardous [ 74 ]. Current industry-wide standards are insufficient to capture the full breadth and depth of PGHD and patient preferences. New standards are needed to represent patient preferences related to communication, care goals, acceptance of treatment and management options, and preferred decision-making styles [ 59 ].

Second is inadequate use of interoperability standards to support consumer-mediated exchange of patient-centered data. As noted, there have been notable uses of the FHIR standard to support the collection and exchange of these data. However, no technical infrastructure yet enables patients to seamlessly collect and share their data across different platforms or different care teams. PGHD pose unique challenges for consumer-mediated exchange. Wearable devices and mobile devices can capture large volumes of data, particularly devices that perform continuous monitoring (e.g., capturing data over regular intervals). But even if these data can be collected in a standardized way, there are significant challenges in aggregating and exchanging the data in a way that is interpretable and meaningful for healthcare decision-making. Clinicians must also be able to identify the source of PGHD (data provenance) to support clinical decision-making.

Third is the lack of guidelines for consumer-facing apps, both in terms of the standard capture and interoperable exchange of data as well as standardized privacy and security practices. Currently, the U.S. has no requirements for consumer-facing apps to store data in standardized ways. Thus, wearable device and app developers have no incentive to standardize data capture in a way that facilitates patient-centered data exchange. Globally, some countries like the United Kingdom (U.K.) have established criteria to assess the interoperability of apps (focused on ensuring that data can be exchanged with EHRs) as part of regulatory oversight, but these criteria do not extensively speak to standardized data capture [ 75 ]. In terms of privacy and security, the Health Insurance Portability and Accountability Act (HIPAA) in the U.S. and the General Data Protection Regulation (GDPR) in the E.U. provide standards for the sharing of health data, including HIE [ 76 77 78 ]. However, as the use of consumer-facing apps continues to grow, there will likely be a need to establish additional privacy standards.

### 4.3. Trending Technology: Supporting Consumer-Mediated Exchange

Progress in the uptake of patient portals and patient apps on mobile phones is good news in the effort to enhance consumer-mediated HIE.

#### 4.3.1. Current Trends

PHRs have been established and used not only in the U.S., but also in other Organization for Economic Co-operation and Development (OECD) member countries, such as Australia and member countries of the E.U.

Globally, trends in PHR use are mixed. A review of national-level PHR use in advanced countries founds that in Finland, 53% of the subject population had accessed the patient portal by the end of 2017. In Sweden, more than 3 million users had accessed online records as of 2019 (out of a total population of approximately 10 million). These trends indicate high use of PHRs. However, the number of downloads of the National Health Service (NHS) App was only approximately 200,000 in the U.K. as of early 2020. The proportion of the population that used a patient portal at least once a month was less than 5% in Denmark and less than 1% in Estonia and Australia [ 79 ].

Trends in PHR use in the U.S. offer encouraging insights into patients' interest in downloading their health information. Analysis of the nationally representative 2022 U.S. Health Information National Trends Survey (HINTS) reveals a growing trend in the percentage of patients who were offered and subsequently accessed a patient portal, with a 46% increase in patient portal access observed from 2020 to 2022 [ 80 ]. The HINTS data also show a positive association between younger age, white race, and higher socioeconomic status with accessing a patient portal at all, consistent with the results of previous research [ 81 ].

In 2019, almost three-quarters (70%) of U.S. hospitals facilitated patient access to their health information through apps—up from only half (just over 50%) the previous year. There was a concomitant increase in 2019–2020 in the number of apps integrated with EHRs that catered to patients and clinicians, with the total number of unique apps in the five leading EHR app galleries increasing from 600 to 734 [ 82 ]. Similar trends in the use of mobile health apps by hospitals and healthcare institutions are being seen globally [ 83 ].

In addition to being standalone (e.g., in mobile phones), patient apps can be embedded in health IT systems through EHRs and PHRs, as shown in Figure 1 . Use of these apps has the potential to increase patient engagement in their own healthcare and healthcare planning. In addition, such use may give clinicians a more comprehensive view of patients' health and well-being, though exploration of how these data should be combined and presented to clinicians still needs to be considered [ 84 ]. While further research is needed to understand the impact of patient apps, involving patients in their own care may enable them to shape that care; and empowering patients to tailor treatments to fit with their needs and preferences has potential to result in improved health outcomes [ 85 ].

Standard-based APIs play a crucial role in facilitating bi-directional data exchange between patient apps and care organizations' EHRs. As noted earlier, the ONC Cures Act Final Rule requires secure access to patient health information through standard-based (HL7® FHIR®) APIs. As a result of the supportive regulatory and policy landscape, the incorporation of FHIR-based APIs has been growing, further driven by recognition that this advance provides a technical solution to interoperability for both federal and non-federal stakeholders [ 26 ]. This trend reflects a concerted effort to streamline and improve the efficiency of health data access and exchange, aligning with the broader goal of enhancing healthcare interoperability in the U.S. A recent survey by HL7 on the global adoption of FHIR standards indicate that many countries are adopting these standards and most respondents noted that FHIR adoption will only continue to increase [ 86 ].

#### 4.3.2. Key Challenges

Four main technical challenges are likely to confront the field as it moves toward patient-centered HIE. First, adoption of standard-based APIs still varies across health systems. According to an ONC data brief—based on trends in US hospital API use from the 2020 and 2022 American Hospital Association IT Supplements survey—87% of hospitals are using APIs to support patient access to health information through apps. Additionally, 60% of hospitals allow patients to submit data through APIs [ 87 ]. But despite the increasing use of standard-based APIs to facilitate exchange of patient data, disparities remain based on hospital size and system affiliation. Additionally, the incentive structure of healthcare is a significant driver of API adoption, or lack thereof. For example, APIs are a key enabler of clinical data exchange in value-based care models, but a lack of trust between providers and payers can limit their use [ 88 ].

Moreover, while information can be exchanged between apps and EHRs, a notable gap exists in the integration of patient-contributed data into the EHR—lack of write capability [ 86 , 89 ]. Patient-facing apps, in particular, are largely limited to read-only (except for low-risk, tightly constrained write functions like scheduling). As a result, data submitted by patients through apps are not being seamlessly integrated into the formal EHR. More information is needed about the types of PGHD best suited for EHR integration as well as strategies for addressing integration challenges. There is also a need for more research to assess the impact of integrating PGHD from patient apps into EHRs, including if and how it promotes patient-centered care across diverse care settings [ 90 ]. It is notable that EHR vendors typically exhibit reluctance to fully integrate patient-contributed data into an EHR, restricting the capabilities of the write APIs that do exist [ 26 ].

Second, the full adoption of FHIR, recognized as a standard for representing health data, faces challenges. FHIR has multiple versions, is still in a maturing phase, and its adoption, while gathering momentum, is not widespread throughout the healthcare industry. However, recent data from the ONC reveal that more than two-thirds of hospitals reported using HL7® FHIR® APIs to enable access in 2022. This indicates growing acknowledgment and utilization of FHIR APIs, signaling a potential shift towards broader adoption. Despite the challenges, the increasing recognition of FHIR's importance and the reported use in enabling access suggest a positive trajectory in the integration of standardized APIs in the health data exchange ecosystem [ 88 ]. Globally information and communication technology (ICT) vendors also recognize FHIR as an important part of the healthcare market [ 91 ]. Several countries, notably Australia, Canada, Switzerland, and Denmark, have adopted and published national implementation guidance for FHIR [ 92 93 94 ].

Third, clinicians are raising burden, liability, and utility concerns over the integration of patient-contributed data. Burden concerns focus on the volume of data clinicians would need to review. Liability concerns include the possible inadvertent overlooking of data in their care organization's system that, in retrospect, could have been reviewed and acted upon to potentially improve a clinical outcome [ 26 ]. These concerns highlight the delicate balance between leveraging valuable patient-contributed data and managing the practical challenges associated with their integration.

Clinicians, care organizations, and patients have differing views on the integration of PGHD in clinical workflow [ 12 , 90 , 95 ]. Clinicians and care organizations question both the utility of PGHD for clinical decision-making and the shifting expectations for the provider-patient relationship. Patients tend to view PGHD use as routine and important for diagnosis, generally, rather than information to be used only when the clinician determines it is clinically relevant. These issues are not unique to APIs; they extend to the broader issues of integrating PGHD into EHRs. Achieving a seamless integration that enhances clinical decision-making without overwhelming clinicians remains a complex task that requires ongoing collaboration between healthcare stakeholders and technology developers.

Fourth, immature data provenance rules contribute to clinician anxiety surrounding write APIs and clinician use of external data [ 26 ]. Data provenance rules need to be improved to successfully enable interoperable exchange. All data in the EHR need associated metadata stored and visible that identify the source system and person/party responsible for the data creation. Data should also include a digital signature or other indicator that records any data alterations (i.e., date, time, responsible party) and specifies the altered field(s), versus indicating simply that a change was made to a section or page. This level of detail will assist clinicians and care organizations in deciding which information to trust and help protect against unauthorized alteration.

## 5. Discussion and Recommendations

Great strides have been made in public policies, standards adoption, and technology that support consumer-mediated HIE. However, additional opportunities in all three areas exist to further its advancement, requiring engagement of multiple stakeholders including informaticians, policymakers, researchers, health systems, clinicians, standards development organizations, patient representatives, and EHR and app developers. Given the heterogeneity in how health systems are organized and in how care is paid for in different countries, a one-size-fits-all approach is not possible—additional study and analyzes will help propose and tailor potential solutions.

### 5.1. Public Policy and Initiatives

#### 5.1.1. Increasing patients' ability to access and share their health information while maintaining privacy

In some countries, including the U.S., patients typically receive healthcare from multiple clinicians working in multiple health systems, which may not use the same EHR and may not share electronic health information in an interoperable way. In addition, there is a growing interest amongst patients to share data they generate outside the clinical setting with their clinicians and care teams. There are opportunities to institute policies and initiatives to ensure that apps leverage FHIR-based APIs, adhere to security and privacy standards, and clearly convey how patient data will be used. In addition, there is an increasing need to empower patients about their own rights. Today, there are growing concerns that when patients are choosing and using apps, they may not have clear and understandable information regarding how the apps will protect the confidentiality of their data and how the third-party's terms of use or privacy policy might permit the company to use or disclose the patient's information to others without seeking further, specific approval from the patient. For example, to help build confidence among patients and across the healthcare market that privacy and security concerns are being addressed, one potential area of exploration would be to develop an “App Developer Code of Conduct”. The adoption of an industry code of conduct among app developers could improve the information available to stakeholders to help inform their decision-making around app use and provide a rubric against which apps could be evaluated. For example, the CARIN Alliance Code of Conduct Accreditation Program (CCCAP) aims to further advance best practices in consumer-mediated exchange, while always protecting the security, privacy, and confidentiality of patient data [ 96 ]. In addition, initiatives like the Argentinian Citizen Digital Health Portal, which informs and empowers patients about their rights and also serves as a personal privacy manager for patients to allow access to their data and review access logs, may serve as an interesting example for others to follow [ 97 ]. Additional research on patients' perspectives and on effective policies and best practices regarding the protection of patient privacy and security of patients' health information—particular for those data generated and directly shared by patients—is imperative for wider adoption of consumer-mediated HIE.

#### 5.1.2. Developing a minimum set of criteria and vetting of apps

In today's API environment, there is a wide range of vetting procedures to guarantee the safety and security of electronic health information shared via apps. In some cases, EHR vendors make available sandboxes for testing apps prior to go-live, and then apply stringent testing procedures prior to app release. The more robust testing is typically reserved for provider apps; less stringent testing seems to be the norm for apps used to facilitate patient data access. Robust and transparent vetting rubrics for all apps would benefit users and developers alike—applying best practices to the review process and ensuring that people know how apps are interacting with and protecting their data. Several federal resources and tools in the U.S. are available to assist app developers to better understand the privacy and security laws that may apply to their app [ 98 99 100 ]. Additionally, ONC's EHR certification program provides a precedent for programmatic review of health technology [ 101 ]. Continued attention to and expansion of these testing protocols and vetting tools will be necessary as the technology landscape continues to evolve.

### 5.2. Standardizing key patient-contributed data

Additional standards are needed to support the collection and exchange of patient-centered data. As wearable devices become increasingly common, there is a pressing need to address gaps in the standardization of the PGHD from these devices. These data have the potential to support whole person care; and require standardized approaches to capture, exchange, and visualize the data. In the U.S. and globally, there is a need for further development of terminology standards and implementation guidelines to support the standardized capture and exchange of PROs and patient preference data. Existing terminology standards do not capture the full range of PROs and patient preferences. Standards for capturing patient preferences around use of data, receipt of laboratory results, accessibility, and use of IT-enabled support tools are particularly lacking [ 59 ]. Standards development organizations should address the gaps, in partnership with patient partners and other stakeholders, as well as explore the further use of the FHIR standard to mediate the exchange of these data. In addition, once standards are developed, public policies are needed to support their adoption and use. Future policy efforts in the U.S. and globally will be needed to ensure that FHIR implementation guides include elements for patient-centered data to provide a supportive policy landscape.

### 5.3. Technology

#### 5.3.1. Enhancing PGHD integration into EHRs

To improve the integration of PGHD into EHRs, there is a need to advance write capabilities, and FHIR implementation guides need to be expanded to include write access. The development of the write implementation guides will require participation of different stakeholders, including clinicians, informaticians, patients and caregivers, policy-makers and standards development organizations. There is also a need for more research and evidence on the optimum approaches for integrating PGHD into EHRs [ 91 , 102 , 103 ]. Several studies indicate that complete implementation and adoption of FHIR standards are needed to improve the ability of PGHD to be integrated into the EHR which in turn has the potential to increase the use of PGHD in clinical decision-making and patient-centered care.

#### 5.3.2. Demonstrating the utility of PGHD for clinical decision-making and improving outcomes

Further research is needed to assess whether inclusion of PGHD improves clinical decision-making, patient-centered care and has the potential to improve health outcomes. Ideally this would take a use case-driven approach, starting with less complicated write functions and gradually moving to more complicated functions. A number of potential use cases could improve clinician decision-making and patient contributions to the medical record, such as writing simple documents to the EHR from third-party apps; implementing an automated method of incorporating data on a patient's daily exercise activities in the form of a concise, textual summary of monthly accomplishments, rather than sending daily raw step counts to the EHR; writing questionnaire responses back into the EHR (e.g., smoking cessation questionnaires) and better leveraging PGHD and PROs data collection; and developing a patient-facing app that allows patients to contact their clinicians and request edits to their record (e.g., medication lists) to support patient data corrections. Building the evidence base will require implementation of a prioritized set of use cases and conducting research studies to assess the impact of PGHD on clinical care and patient outcomes.

#### 5.3.2. Dealing with data provenance and liability

The rules governing capture and representation of data provenance in EHRs should be improved to successfully enable interoperable data exchange. Developers of these rules should carefully consider whether external data (such as patient-contributed data) should be shown separately; merged with native data; or combined with native data but with a distinguishing appearance (e.g., different color or icon). Likewise, there are technical, policy, and legal issues about whether and how data from external sources are validated. For example, an EHR could display external data in a different color until the data are validated. If there are technical or semantic issues in importing/merging data, any resultant implications should be identified and displayed to EHR users. These types of issues could be surfaced by allowing developers to test apps in a sandbox environment, ensuring access to transparent and consistent vetting programs and procedures.

#### 5.3.3. Managing the volume and meaningful synthesis of patient-contributed data

One possibility to address issues related to volume and synthesis of patient-centered data is to develop apps to filter and summarize raw PGHD and PROs data before they reach the clinician. Future EHRs could be made to function like data repositories that provide concise and customizable PGHD and PROs information and specifically relevant or summarized data to providers at the point of care (e.g., via dashboards). This kind of automated system for PGHD and PROs summary and presentation would be preferable to the burden associated with manual review of real-time or near real-time PGHD and PROs data—thereby facilitating the appropriate use of these data in clinical decision-making.

## 6. Conclusions

The current landscape in the U.S. and globally supports a more patient-centered care model. Ongoing advances in technology and data standards provide the technical infrastructure to empower consumers to electronically exchange their information with different stakeholders in ways not possible just a few years ago. These advancements hold great promise for patients to play a more central role in sharing their information in support of more patient-centered care. Additional research and analyzes along with public policies are needed.
